# Comparative Design Study for Power Reduction in Organic Optoelectronic Pulse Meter Sensor

**DOI:** 10.3390/bios9020048

**Published:** 2019-03-29

**Authors:** Fahed Elsamnah, Anubha Bilgaiyan, Muhamad Affiq, Chang-Hoon Shim, Hiroshi Ishidai, Reiji Hattori

**Affiliations:** 1 Department of Applied Science for Electronics and Materials, Kyushu University, Fukuoka 816-8580, Japan; fahed.elsamnah.700@s.kyushu-u.ac.jp (F.E.); misran.muhamad.458@s.kyushu-u.ac.jp (M.A.); 2 COI STREAM, Center for Organic Photonics and Electronics Research (OPERA), Kyushu University, Fukuoka 819-0395, Japan; anubha.bilgaiyan@adachilab.com (A.B.); shim@gic.kyushu-u.ac.jp (C.-H.S.); 3 Hiroshi Ishidai, Konica Minolta, Inc., Ishikawa-cho, Hachioji 192-8505, Japan; hiroshi.ishidai@konicaminolta.com; 4 Global Innovation Center (GIC), Kyushu University, Fukuoka 816-8580, Japan

**Keywords:** organic optoelectronic device, pulse meter, biosensor, photoplethysmogram (PPG)

## Abstract

This paper demonstrated a new design structure for minimizing the power consumption of a pulse meter. Monolithic devices composed of a red (625 nm) organic light-emitting diode (OLED) and an organic photodiode (OPD) were fabricated on the same substrate. Two organic devices were designed differently. One had a circle-shaped OLED in the center of the device and was surrounded by the OPD, while the other had the opposite structure. The external quantum efficiency (EQE) of the OLED and the OPD were 7% and 37%, respectively. We evaluated and compared the signal-to-noise ratio (SNR) of the photoplethysmogram (PPG) signal on different parts of the body and successfully acquired clear PPG signals at those positions, where the best signal was obtained from the fingertip at a SNR of about 62 dB. The proposed organic pulse meter sensor was operated successfully with a power consumption of 0.1 mW. Eventually, the proposed organic biosensor reduced the power consumption and improved the capability of the pulse meter for long-term use.

## 1. Introduction

Wearable health-monitoring devices can promote public health by facilitating a person’s health assessment based on their personal vital signs. Due to considerable increased concern for the monitoring of vital signs in normal conditions as well as during physical activities, there has been an enhancement of wearable medical devices and their performance for personal health care [1,2]. Wearable medical devices have been developed to constantly monitor human performance both comfortably and noninvasively [3,4]. These devices have different forms and target different healthy people or patients based on biosensors such as portable electrocardiogram (ECG) [5], cuff-less blood pressure measurement [6], wearable devices for the diagnosis and therapy of movement disorders [7] and pulse oximeters based on photoplethysmogram (PPG) [8]. The PPG signal is one of the crucial biosignals used for monitoring and evaluating the heart rate (HR) and peripheral oxygen saturation (SpO_2_). The pulse oximeter can be a very useful tool in monitoring and detecting certain disorders such as sleep apnea, which is detected by measuring the SpO_2_ in the blood [9]. Currently, there are various kinds of commercial pulse meters and pulse oximeter devices available on the market. However, there are still some issues with these kinds of pulse meters that need to be improved in areas such as power consumption, signal quality, and the flexibility of the device. In order to make a flexible pulse meter, an organic light-emitting diode (OLED), and an organic photodiode (OPD) are used with the advantage of its design freedom. Moreover, the relatively low cost, simple fabrication, and lower power consumption of the OLEDs and OPDs makes them preferable options for wearable pulse meters [10].

**Figure 1:**
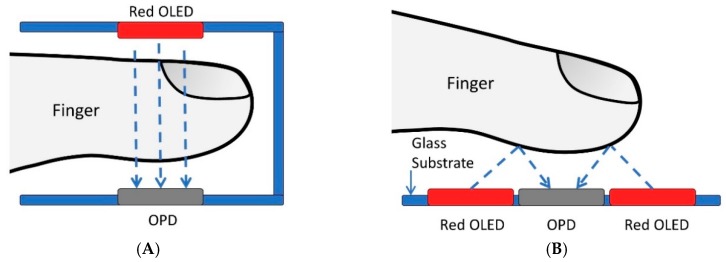
Obtaining the photoplethysmogram (PPG) signal based on an organic pulse meter: (**A**) Transmissive mode; (**B**) Reflective mode [11].

To obtain a PPG signal from a pulse meter, there are two approaches that can be used: a reflective mode and a transmissive mode, as shown in Figure 1, which illustrates the differences between the two methods for an organic pulse meter.

In the transmissive mode, where the light source and the detector are placed on opposite sides as shown in Figure 1A, the light from the OLED goes through the finger and is received on the OPD from the other side. This mode is limited to be applied on terminal body parts such as fingertips and ear lobes. On the other hand, the reflective mode, which is illustrated in Figure 1B, uses light reflection instead of tissue transillumination. The light source and the detector are both mounted side-by-side, where the OPD detector obtains the reflected light from the human skin. Therefore, by taking advantage of the design freedom presented by the organic materials and fabricating it onto a flexible substrate, the pulse meter can be easily worn or attached to the human body. However, in the pulse oximeter, it is difficult to perform accurate oxygen saturation level measurement on some body locations, such as the torso or limbs, using the reflection mode due to the higher density of fat and lower density of blood vessels in those locations [12]. Several researchers have started to work on the reflectance detection method [13,14], as the transmissive mode has been reported to be uncomfortable and have a limited usage. The reflective mode, on the other hand, has several advantages over the transmissive method, as it can be applied to different parts of the body, not only on the fingertips or the thin portion of the ear, to measure the PPG signal.

One of the best advantages of the organic optoelectronic devices is the freedom of design, where the designer engineer can make the best match between the OLED and OPD for improving the power consumption and the PPG signal quality. There are several proposed design structures in the literature. In [15], separated two square-shaped OLED devices and one rectangular-shaped OPD device were proposed as a transmissive mode pulse oximeter, while in [16], they proposed a monolithic device of array square-shaped OLEDs and OPDs for measuring the oxygen saturation and creating two-dimensional (2D) oxygenation maps. The configuration of an annularly-shaped photodiode (PD) ring and light-emitting diode (LED) located in the center was first proposed by [12] for improving the battery longevity of the inorganic reflective pulse oximeter. In [17], a circular-shaped OPD in the center of half-ring-shaped of red polymer light-emitting diode (PLED) and the second half-ring for green PLED design was proposed for the flexible reflective pulse oximeter. Also, a ring-shaped OPD surrounding a circular-shaped OLED was proposed in [18] for a flexible pulse oximetry sensor. They employed an optical simulation for deciding the best dimension area and the best distance between the OLED and the OPD for low-power consumption. However, the PPG signal quality was not adequately addressed in the previously proposed designs. Moreover, the dimension design, the material structure, and the characteristics of the OLED and OPD in this work are different than those in previous works. Consequently, we proposed and compared different dimensions and material structures based on our previous works in [11,19] as part of our continuous research into improving the power consumption and the signal quality of portable pulse meters, and highlighted the significance of the optical simulation in designing the OLED and the OPD.

There are several studies that have been carried out in the literature to improve the power consumption of the pulse meter. In [20], they proposed a reflective pulse meter prototype with a total power of about 40 mW, with the LED driving current between 17.4–50 mA. In contrast, [21] proposed an analog single-chip pulse oximeter that was implemented with 4.8 mW of total power consumption required of the LED power and the processing power chips. A fully integrated pulse oximeter front-end with about 1 mW of total power consumption, 0.31 mW for the LEDs, and 0.53 mW for the front-end was proposed in [22]. For improving the flexible pulse oximeter, a promising result of the average OLED’s power consumption of 0.097 mW, at a full-duty cycle, was reported in [18]. While we are presenting the power consumption of the previous works on the pulse oximeter, which requires two light sources, it is worth mentioning that our proposed pulse meter requires a single light source that leads to half the power consumption of two light sources when they operate at the same time with a full-duty cycle.

The PPG signal can be contaminated by noises from different kinds of sources such as motion artifacts, electronic noises, and high-frequency noises from the ambient light fixture and appliances. Consequently, extensive works have been done on denoising the PPG signals with several noise reduction techniques such as discrete wavelet transform (DWT) [23], independent component analysis (ICA) [24], and morphological characteristics comparison [25]. In [26], sporadic noise was reduced from a continuous periodic signal after applying a cluster analysis for picking similar replications or pulses from a periodic single, while in [27], a pattern recognition filtering system for PPG and ECG signals was proposed. However, it is difficult to implement these methods into wearable devices due to their requirements and complexity. In this work, we implemented a finite impulse response (FIR) digital filter in order to improve the signal’s quality due to its efficacy in the embedded systems.

This paper presents the significance of designing an effective OLED and OPD structure that is guided by optical simulation for improving pulse meter sensors in terms of power consumption and signal quality. Two new designs of OLED and OPD were fabricated and tested for their performance verification. The rest of this paper is organized into three sections. Section 1 is the introduction. The materials and methodology are present in Section 2, where we discuss the approach that we used to simulate the new design, define the characteristics of the organic optoelectronic device along with explaining the driving circuit, and describe the digital filter that we applied to the system. In Section 3, we discuss the comparative results of the two devices as well as the results of the obtained PPG signals from different parts of the body.

## 2. Materials and Methods

Two different design structures were fabricated and named as Device-A and Device-B. In Device-A, a circle-shaped red OLED was set in the center of the pulse meter with a total emitting area of 0.03 cm^2^ and surrounded by a ring-shaped OPD with a surface area 0.16 cm^2^, whereas in Device-B, a circle-shaped OPD was set in the center and surrounded by a ring-shaped red OLED with areas of 0.03 cm^2^ and 0.16 cm^2^, respectively. The rationale behind the use of the ring-shaped OPD structure that surrounds the OLED is to allow the OPD to collect the reflected photons from the skin efficiently. The proposed devices were tested on healthy individuals, who gave their informed consent for inclusion before they participated in the study.

### 2.1. Optical Simulation

**Figure 2:**
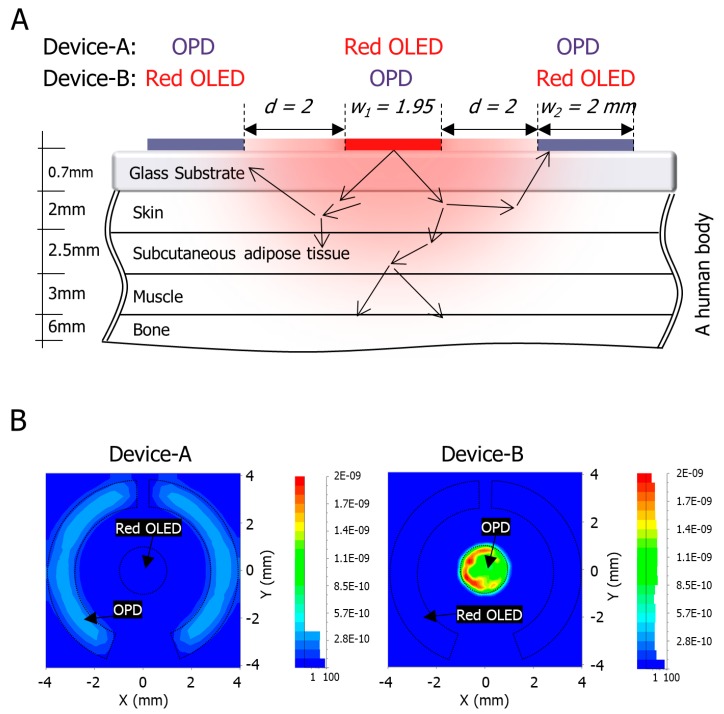
(**A**) Optical skin schematic model of a human finger, an organic light-emitting diode (OLED) as a light object source, and an organic photodiode (OPD) as a surface receiver, where *d* is the distance between the edges of the OLED and the OPD, *W*_1_ is the diameter of the OLED, and *W*_2_ is width of the OPD; (**B**) The optical simulation results at the surface receiver.

In order to get the best OLED and OPD design for improving the power consumption and quality of the signal, a simplified finger model and OLED/OPD model were simulated optically by LightTools software (Synopsys, Inc., California, USA). The simulator traces the light rays using the ray-tracing method in combination with the Monte Carlo method, where two opposite design structures of the pulse meter were simulated: Device-A and Device-B. The finger model was simplified into a four-layer structure of skin, subcutaneous adipose tissue, muscle, and bone. The optical parameters of these layers were approximated from the literature in [28,29,30,31]. The thickness of each layer was approximately assumed as 2 mm, 2.5 mm, 3 mm and 6 mm, respectively. The distribution of the light rays from the OLED into the human body and the reflection on the OPD were illustrated in Figure 2A. The pulse meter model was composed of a light object source (625 nm) that represented the red OLED, and a surface receiver that represented the OPD.

The light ray’s energy is determined, in the optical simulation, by the source’s power and the number of rays emitted. The radiant powers of the light object source of Device-A and Device-B were assumed, based on our previous OLED device, as 1.4 μW and 7.5 μW, respectively. The number of the traced rays were 1,000,000 rays. According to the simulation results in Figure 2B, the maximum estimated irradiance of Device-A and Device-B were 3.7 × 10^−10^ W/mm^2^ and 2 × 10^−9^ W/mm^2^, respectively. By multiplying the irradiance with the area of the OPD, the total power of Device-A will be 5.9 × 10^−9^ W and that of Device-B will be 6 × 10^−9^ W, where the maximum irradiance was assumed to be distributed equally, which is expected to provide closer values of the PPG amplitude from both OPD devices. Therefore, from these two opposite design structures, we conducted a comparison in order to choose the best one in terms of power consumption and signal quality.

### 2.2. The Organic Optoelectronic Device

**Figure 3:**
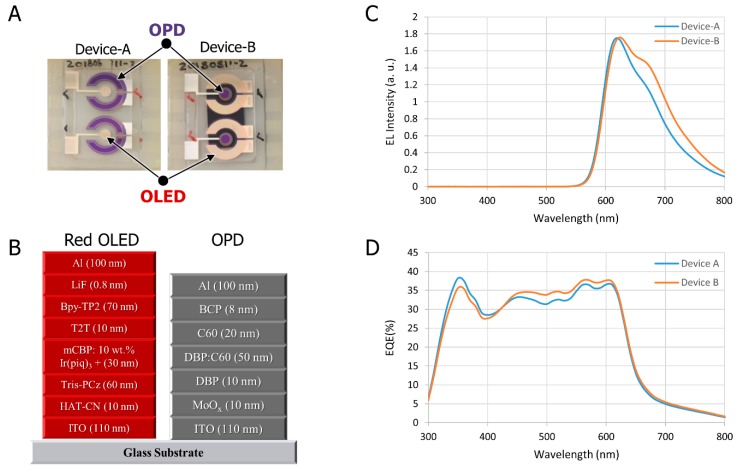
(**A**) Top view of the fabricated optoelectronic Device-A and Device-B; (**B**) The organic device structure of the OLED and OPD; (**C**) Electroluminescence spectrum of the OLED with respect to the wavelength (nm); and (**D**) External quantum efficiency (EQE) of the OPD with respect to the wavelength (nm).

In this work, we fabricated two different pattern monolithic devices, Device-A and Device-B. The red OLED and the OPD were placed on the same 0.7 mm thick glass substrate, as shown in Figure 3A, where the two-identical ring-type OLED/OPDs were fabricated on the same substrate as a spare and reference device. The red OLED was adopted in this work due to its various advantages. For instance, the green OLED light is efficiently absorbed through the skin, which leads to producing a higher signal-to-noise ratio (SNR) level than the red OLED. However, absorbing more light through the skin, especially darker skin where the shorter wavelengths of light are strongly absorbed by melanin [32], limits the depth that light can pass through, and hence weakens the strength of the PPG signal [33]. Based on that, green OLEDs will face some challenges when they are used as a light source for wearable pulse oximeters.

The performances of the OLED and OPD devices are essential to the quality of the pulse meter measurements. We used the same material structure of the OLED in Device-A and Device-B and the same material structure of the OPD in both devices. In the fabrication process, we prepared 2.5 cm × 2.5 cm glass substrates coated with indium-tin-oxide (ITO) and cleaned via sonication in detergent, deionized (DI) water, acetone, and isopropyl alcohol (IPA). Next, the substrates were boiled in IPA for 10 min and subjected to UV-ozone treatment for 15 min. Then, the samples were loaded into a thermal evaporator chamber where the organic and metal layers were deposited with proper shadow mask patterns for the OLED, followed by the OPD. After the evaporation processes, the samples were encapsulated in a glove box by glass lids using UV curable epoxy resin [19]. The device structure of the OLED was composed of ITO (110 nm)/HAT-CN (10 nm)/Tris-PCz (60 nm)/mCBP:10 wt.% Ir(piq)3 + (30 nm)/T2T (10 nm)/Bpy-TP2 (70 nm)/LiF (0.8 nm)/Al (100 nm), and the device structure of the OPD was composed of ITO (110 nm)/MoO_x_ (10 nm)/DBP (10 nm)/DBP:C60 (50 nm)/C60 (20 nm)/BCP (8 nm)/Al (100 nm) as shown in Figure 3B. Figure 3C shows the electroluminescence (EL) characteristic of the OLED with respect to the wavelength, where the maximum intensity of light is at 625 nm. OLEDs have advantages over LEDs such as high flexibility and low manufacturing cost. The very thin organic layers of the OLED and OPD device make it applicable for fabrication onto a flexible substrate, which will be comfortable when placed on the human skin. The EQE of the OPD device at 625 nm, of a zero-bias condition, for Device-A and Device-B was about 37%, which is compatible with the red OLED wavelength, as shown in ‎Figure 3D.

### 2.3. The Driving Circuit

**Figure 4:**
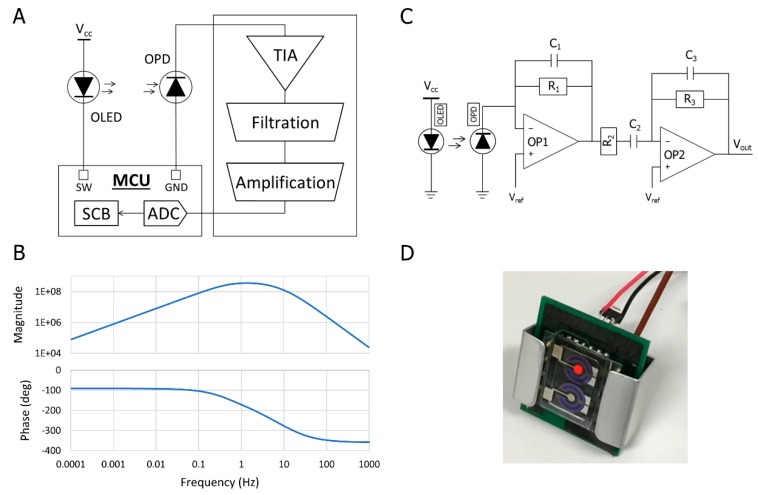
Circuit design of the pulse meter. (**A**) The system structure of the pulse meter; (**B**) The Bode plot of the bandpass filter (BPF) circuit; (**C**) The proposed analog circuit for acquiring the photoplethysmogram (PPG) signal from the pulse meter; and (**D**) The proposed portable pulse meter prototype.

The system structure of the proposed pulse meter is composed of three parts: an optoelectronic device, an analog circuit, and a microcontroller unit (MCU). The analog circuit employed in this work involved three main stages: a transimpedance amplifier (TIA), a bandpass filter (BPF), and amplification, as illustrated in Figure 4A.

The generated photocurrent from the OPD, as a response of the reflected light from the blood vessels, is a very small amount in the nanoampere range, which requires a current-to-voltage converter such as a TIA to translate the current output of the OPD to a voltage signal in the first stage of the analog circuit. The TIA should have a low input impedance and low output impedance to avoid impedance mismatch in the circuit. It offers a low impedance to the OPD, and isolates it from the output voltage of the operational amplifier [34,35]. The next stage is the analog filtration process where the PPG signal needs to be filtered out before the amplification. The PPG signal contains an alternating current (AC) component and a direct current (DC) component where the aim is to eliminate the DC component and amplify the AC component. Therefore, a BPF that was composed of a low-pass filter to eliminate the high-frequency noise coming from the ambient light fixture and AC devices, and a high-pass filter to eliminate the DC signal, was applied with a cut-off frequency from 0.5 Hz to 16 Hz. The transfer function (TF) of the BPF is stated in Equation (1), which indicates three negative poles of a stable system where we can simulate the frequency response of the circuit, as shown in Figure 4B:

(1) $$H\left(S\right)=\frac{SC_{2}R_{1}R_{3}}{\left(1+SC_{1}R_{1}\right)\left(1+SC_{2}R_{2}\right)\left(1+SC_{3}R_{3}\right)}$$

The last stage after filtering the signal is to amplify it to be suitable for the analog-to-digital converter (ADC) process in order to send the data via a serial communication block (SCB) or Bluetooth low energy (BLE). Therefore, in order to amplify the signal in the range of 0 to Vdd for the single-supply op-amp, a virtual ground (Vref) needs to be connected, as shown in Figure 4C. The MCU module that we applied in this work was a PSoC4 CYBLE-214015-01 module. It improved the battery life by offering low-power modes, during which the chip offered restricted performance for the lowest possible power consumption. The final prototype of the wearable pulse meter is shown in Figure 4D.

### 2.4. Digital Filter

**Figure 5:**
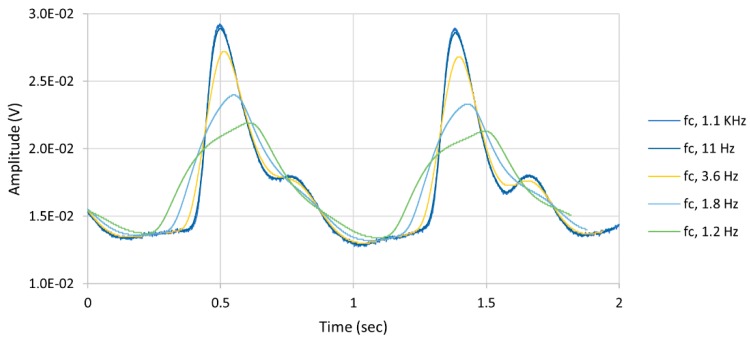
Digital low-pass filter effect on the PPG signal, where the signal lost its morphological structure if the cut-off frequency (*f_c_*) was set very close to the carrier frequency.

In portable pulse meters, the PPG signals are susceptible to corruption by noise and motion artifacts, where the analog filter is not adequate to eliminate these noises. Hence, the digital filter was implemented to improve the PPG signal’s quality. There are several noise reduction methods using digital signal processing techniques in the literature such as the discrete wavelet transform (DWT) [23], morphological characteristics comparison [25], and cluster analysis [26]. However, it is difficult to implement these methods into embedded systems due to their requirements and complexity. Therefore, we implemented a finite impulse response (FIR) digital filter in order to improve the signal’s quality. The representative equation of the FIR digital filter is shown in Equations (2) and (3):

(2) $$y\left[n\right]=\overset{L}{\underset{k=0}{\sum }}b_{k}x\left[n-k\right]$$

(3) $$b\left[k\right]=\{\begin{array}{c} \begin{array}{cc} \alpha +(1-\alpha )\cos\left(\frac{2\pi k}{N}\right) & -N\leq k\leq N \end{array}\\ \begin{array}{cc} 0\;\;\;\;\;\;\;\;\;\;\;\;\;\;\;\;\;\;\;\;\;\;\;\;\;\;\;\;\;\;\;\;\;\;\;\;\;\;\;\; & elsewhere \end{array} \end{array}$$

where *y*[*n*] is the output sequence of the digital filter; *x*[*k*] is the input sequence; L is the interval; *b*[*k*] is the weight coefficient; and α is the Hamming window coefficient 0.54 [36,37]. The cut-off frequency of the FIR digital filter was set at 16 Hz, as choosing a very low cut-off frequency would alter the PPG waveform structure and lose information from the signal such as the systolic peak, dicrotic notch, and diastolic peak, as shown in Figure 5. The FIR digital filter reduced the noise and enhanced the stopband slope from −20 dB/decade in the analog filter to more than −86 dB/decade.

### 2.5. SNR Measurement

The common term for quantifying the digital signal is the signal-to-noise ratio (SNR), and it can be simply calculated, as formulated in Equation (4). Fast Fourier transform (FFT) is a fast computation algorithm for discrete Fourier transform (DFT), which is defined by the formula in Equation (5). FFT analysis was used to compute the noise of the digital signal by converting the time-domain signal to the frequency domain by decomposing a sequence of values into components of different frequencies.

(4) $$SNR_{dB}=20log_{10}\left(\frac{A_{signal}}{A_{noise}}\right)$$

where *A_signal_* is the amplitude of the signal and *A_noise_* is the amplitude of the noise.

(5) $$X_{k}=\overset{N-1}{\underset{n=0}{\sum }}x_{n}e^{-i2\pi kn/N}\;,\;\;\;\;\;\;\;\;k=0,\ldots ,\;N-1$$

where *x_n_* is discrete-time input sequence; *X_k_* is the DFT; and *N* is the number of samples.

**Figure 6:**
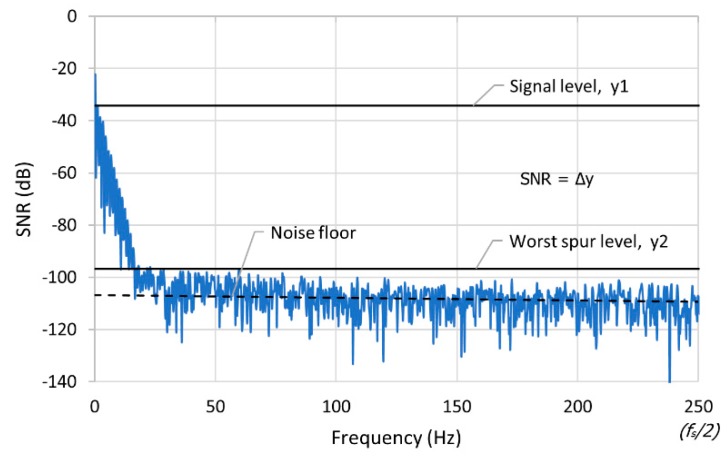
Fast Fourier transform waveform to calculate the signal-to-noise ratio (SNR) of the PPG signal.

The spectra of the FFT is a series of *N/2* points in the frequency domain from 0 Hz to *f_s_/2* Hz, where *N* is the number of samples and *f_s_* is the sampling frequency. The SNR of the PPG signal is calculated by subtracting the amplitude of the PPG signal from the amplitude of the noise level in dB, as illustrated in Figure 6.

## 3. Results and Discussion

### 3.1. Comparative Results for Device-A and Device-B

**Figure 7:**
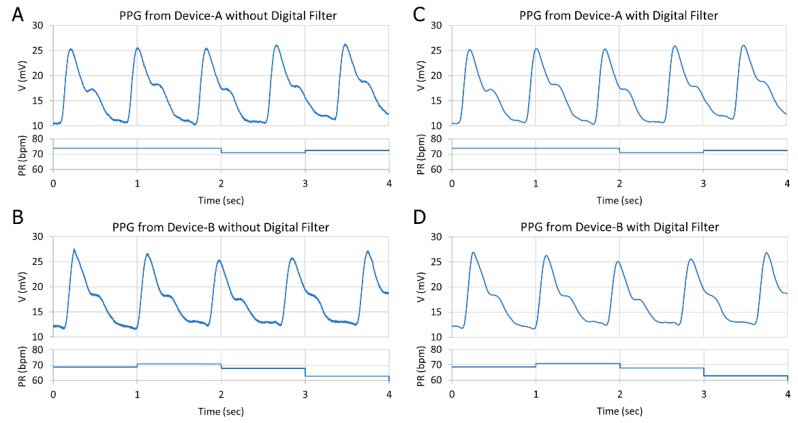
Comparison between the PPG signal from (**A**) Device-A without a digital filter; (**B**) Device-B without a digital filter; (**C**) Device-A after a digital filter; and (**D**) Device-B after a digital filter.

The performance of the proposed pulse meter was tested in vivo on a healthy male subject. The portable pulse meter was attached to the index finger to acquire the PPG signal from Device-A and Device-B sequentially, in order to compare their performance. The PPG signals were obtained from the same subject for a specific time period while he was resting in a chair. For the evaluation process, the data were recorded at the sampling frequency of 500 SPS. The FIR digital filter was applied at a 16 Hz cut-off frequency. The heart rates were extracted accurately in both devices and estimated by averaging the beat-to-beat interval of the peaks in the PPG signal. The portable pulse oximeter (NURSE ANGIE, Custom Co., Tokyo, Japan) was selected as a reference device for estimating the pulse rate (PR) due to its ability to measure the PR in the range of 25 bpm to 250 bpm with a resolution of 1 bpm and accuracy of 3% error. The proposed pulse meter showed accurate results of about 1.5% error of the PR compared to the commercial reference. Figure 7 shows the comparison between the PPG waveform of Device-A and Device-B before and after applying the digital filter. Both devices were reliable and acquired the PPG signal clearly and were almost similar in their output.

A summary of the PPG signal characteristics of Device-A and Device-B are shown in Table 1.

The amplitude V_p-p_ of the PPG signal in Device-A and Device-B was roughly 15 mV, which was as expected in the optical simulation where the amount of reflected light that was received from both OPDs was almost equal. There was a slight difference between the SNR in Device-A and Device-B, which could be due to the difference in the OPD dimension area where the biggest area gained more noise. It is noteworthy that the power consumption recorded a significant disparity between the two devices, where Device-B consumed 8 mW, while Device-A consumed only 0.1 mW. Consequently, these findings support the assumption that designing an OLED to emit a large amount of light is not necessary to produce a high-quality PPG signal; instead, it will consume more power. On the other hand, designing an effective OLED and OPD structure guided by optical simulation led to the best result in terms of power consumption and signal quality. The digital filter demonstrated a noticeable improvement of about 25% in both devices from 45.4 dB to 62.6 dB in Device-A and from 47.4 dB to 63.3 dB in Device-B. As a result, Device-A was selected as the effective device structure in terms of power consumption and quality level in comparison with Device-B.

### 3.2. Results of Device-A on Different Body Parts

**Figure 8:**
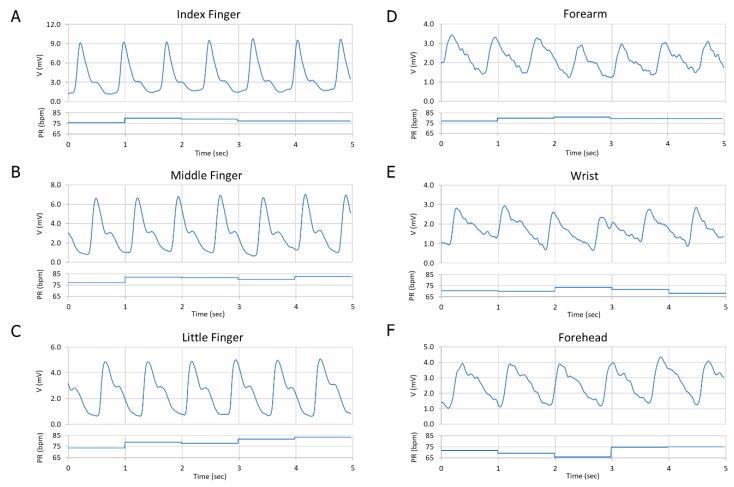
Comparison between the PPG signal on different parts of the body: (**A**) Index finger; (**B**) Middle finger; (**C**) Little finger; (**D**) The forearm; (**E**) The wrist; and (**F**) The forehead.

The Device-A pulse meter was attached to multiple locations on the body (index finger, middle finger, little finger, forearm, wrist, and forehead) within a specific time period. The PPG signals were obtained from those body locations sequentially, from the same subject, in order to compare the PPG signal quality between them, as shown in Figure 8.

Device-A successfully showed a very clear PPG signal on the fingers and a lower quality signal on the forearm, wrist, and forehead. Table 2 summarizes the amplitude, the average pulse rate (PR), and the SNR of the different PPG signals.

The amplitude of the PPG signal varied between 2–20 mV in the devices. There are several factors that can affect changes in the signal amplitude and can be influenced by the type of human skin, the position of the finger on the OPD, and how much pressure is applied from the finger to the OPD where the amount of the reflected light from the arterial blood vessels on the OPD changes based on that pressure. The SNR table demonstrated a very good quality level from the three fingers with an average of 56 dB and showed a good quality from the forehead of 51.4 dB, while it presented a lower quality signal on the forearm and on the wrist position of about 46 dB, as the fat tissue in these parts is thicker, and the arterial blood vessels are deeper than in the skin tissue. In such cases, the intensity of the OLED needs to be increased, which means more power is consumed. For that trade-off between the SNR level and power consumption, we chose a suitable current source for the OLED at 20 μA, 5 V, in order to obtain a very good quality signal from the fingers and a good quality signal from the other body parts. The PR was calculated accurately based on our algorithm. The results showed that the proposed pulse meter based on the new OLED and OPD design structure was capable of acquiring the PPG signals accurately and consistently and was able to minimize the power consumption to as low as 0.1 mW in comparison to the other design structure.

## 4. Conclusions

This paper addressed the significance of designing an effective OLED and OPD structure to improve pulse meter sensors in terms of power consumption and signal quality. Designing an OLED that emits a high amount of light is not necessary to produce a high-quality PPG signal; instead, it will consume more power. On the other hand, designing an effective OLED and OPD structure guided by optical simulation led to the best result in terms of power consumption and signal quality. That assumption was verified by comparing two opposite pulse meter design structures. One had a circle-shaped OLED in the center of the device and was surrounded by a ring-shaped OPD, while the other had the opposite structure. The two methods of obtaining the PPG signal—the reflective method and the transmissive method—were described in this paper, where the reflective method was implemented. The materials and methods that were used in this work, which were the optical simulation, the organic optoelectronic device, the driving circuit, the FIR digital filter, and the SNR measurement, were discussed in detail. The proposed pulse meter successfully showed compatible characteristics, as Device-A effectively demonstrated its ability to measure a clear PPG signal up to a SNR of 62 dB from different parts of the body and operated at a very low power consumption of 0.1 mW, which is essential for the long-term use of portable medical devices.

In future work, the wireless monitoring of the PPG signals will be studied. In order to improve the power consumption performance of the wireless pulse meter, BLE will be integrated into the system. Moreover, the OLED and OPD will be fabricated onto a flexible substrate. These additives will increase the pulse meter’s reliability and flexibility of use as a comfortable wearable device for medical applications.

## Figures and Tables

**Table 1:** Summary of the PPG signal’s quality for Device-A and Device-B.

Sample No.	Average V_p-p_ (mV)	SNR (dB) without Digital Filter	SNR (dB) with Digital Filter	Current Consumption (μA)	Power Consumption (mW)
**Device-A**	15	45.4	62.6	20	0.1
**Device-B**	14.5	47.4	63.3	1600	8

**Table 2:** Summary of the PPG signal’s quality at different parts of the body. PR: pulse rate.

Sample No.	V_p-p_ (mV)	Average PR (bpm)	SNR (dB)
Index finger	8.1	78	58.3
Middle finger	6.0	81	57.2
Little finger	4.2	79	54.4
The forehead	2.9	71	51.4
The forearm	2.0	79	46.9
The wrist	2.3	71	45.7
